# Items

**Published:** 1904-01

**Authors:** 


### ITEMS.

The Palisade Manufacturing Company, Yonkers, N. Y., has issued a handsome brochure entitled, *The Actual Invalid in Tuberculosis,* which is beautifully printed and illustrated by diagrams and charts. It will be sent to any physician upon application to the publishers.

The Fellows Hypophosphite Company, of New York and London, has published a pamphlet entitled, *An Illustration of Therapeutic Conservatism.* It is handsomely printed and contains much material of interest. Medical letters may be addressed to Mr. Fellows, 26 Christopher Street, New York.

The Antikamnia Chemical Company, of Saint Louis, has issued its calendar for 1904, on the obverse of which is the head of a beautiful nun and is entitled, Confidence. On the reverse, in addition to the calendar for the year, is a synopsis of information relating to antikamnia tablets. This souvenir calendar has been sent to every physician in the world. If, perchance, some have failed to receive it, it will be sent upon application to the publishers.

The Oppenheimer Institute has sent out a pamphlet giving a general statement of the history and development of its work, including that pertaining to the Women's National Auxiliary. This document, which displays artistic taste in typography and make-up, may be had upon application to the secretary, 170 Broadway, New York.

The M. J. Breitenbach Company, of New York, are again distributing their ever popular year book. The edition for 1904 is uniform with those of previous years, which enables physicians to preserve records of daily memoranda year by year in volumes that are precisely alike in general make-up and binding. It is really one of the most convenient books for the office table with which we are familiar. The incidental advertising of Gude's peptomangan not only is unobjectionable, but serves to remind physicians of an excellent preparation of iron.

The United States Civil Service Commission will hold an examination at Buffalo, January 27-28, 1904, from which to make certification to fill vacancies in the position of physicians in the Philippines service. Age limit, 20 to 40 years; salary, $1,200 to $1,800. Application should be made to the secretary of the local board at the Buffalo postoffice.

